# The Rise of 1,4‐BN‐Heteroarenes: Synthesis, Properties, and Applications

**DOI:** 10.1002/advs.202200707

**Published:** 2022-04-14

**Authors:** Cheng Chen, Cheng‐Zhuo Du, Xiao‐Ye Wang

**Affiliations:** ^1^ State Key Laboratory of Elemento‐Organic Chemistry College of Chemistry Nankai University Tianjin 300071 China

**Keywords:** azaborines, boron, conjugated materials, heteroarenes, heteroatom, organic semiconductors, polycyclic aromatic hydrocarbons

## Abstract

BN‐heteroarenes, which employ both boron and nitrogen in aromatic hydrocarbons, have gained great attention in the fields of organic chemistry and materials science. Nevertheless, the extensive studies on BN‐heteroarenes are largely limited to 1,2‐azaborine‐based compounds with B–N covalent bonds, whereas 1,3‐ and 1,4‐BN‐heteroarenes are relatively rare due to their greater challenge in the synthesis. Recently, significant progresses have been achieved in the synthesis and applications of BN‐heteroarenes featuring 1,4‐azaborines, especially driven by their significant potential as multiresonant thermally activated delayed fluorescence (MR‐TADF) materials. Therefore, it is timely to review these advances from the chemistry perspective. This review summarizes the synthetic methods and recent achievements of 1,4‐azaborine‐based BN‐heteroarenes and discusses their unique properties and potential applications of this emerging class of materials, highlighting the value of 1,4‐BN‐heteroarenes beyond MR‐TADF materials. It is hoped that this review would stimulate the conversation and cooperation between chemists who are interested in azaborine chemistry and materials scientists working in the fields of organic optoelectronics, metal catalysis, and carbon‐based nanoscience etc.

## Graphical abstract

Boron and nitrogen (BN)‐heteroarenes have gained great attention in organic chemistry and materials science. Recently, significant progresses have been achieved in the synthesis and applications of BN‐heteroarenes featuring 1,4‐azaborine moieties. This review summarizes the synthetic methods and recent achievements of 1,4‐azaborine‐based BN‐heteroarenes, and discusses their unique properties and potential applications, in order to stimulate the synergistic development of azaborine chemistry and materials research.

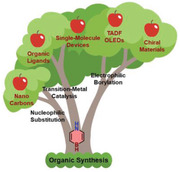

## 1 Introduction

Since the discovery of benzene by Faraday in 1825, monocyclic and polycyclic aromatic hydrocarbons (PAHs) have been attracting wide attention in the fields of organic chemistry and materials science.^[^ ^1^ ^]^ During the past decades, there has been increasing interest in introducing BN units into the backbone of benzene and PAHs to modulate their chemical and physical properties.^[^ ^2^ ^]^ The diatomic polar BN units and nonpolar CC units are isoelectronic moieties while showing great discrepancy in *π*‐electron distribution. The resulting BN‐heteroarenes display unique properties compared with all‐carbon analogs, demonstrating great potential for applications in biological systems, coordinate chemistry, and organic electronics.^[^ ^2^, ^3^ ^]^

**Figure 1:**
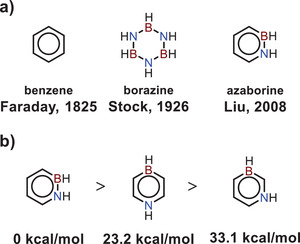
a) Chemical structures of benzene, borazine, and 1,2‐dihydro‐1,2‐azaborine. b) Thermodynamic stability of three azaborine isomers.

In 1926, Stock and Pohland reported the synthesis of borazine,^[^ ^4^ ^]^ which is known as inorganic benzene (**Figure** **1**a). The discovery of borazine has promoted the development of partially BN‐substituted benzene derivatives, namely azaborines. According to the relative positions of embedded boron and nitrogen atoms, azaborines are classified into three isomers: 1,2‐azaborine, 1,3‐azaborine, and 1,4‐azaborine. Theory calculations have revealed that the thermodynamic stability of these three isomers follows: 1,2‐azaborine > 1,4‐azaborine > 1,3‐azaborine (Figure 1b).^[^ ^5^ ^]^ The synthesis of 1,2‐azaborine‐embedded PAHs was pioneered by Dewar and co‐workers in 1958,^[^ ^6^ ^]^ whereas the monocyclic 1,2‐dihydro‐1,2‐azaborine was only successfully synthesized by the Liu group in 2008.^[^ ^7^ ^]^ Up to date, 1,2‐azaborine and its *π*‐extended conjugated molecules have been widely studied and thoroughly reviewed.^[^ ^3^, ^8^ ^]^ By comparison, 1,3‐azaborine displays the lowest thermodynamic stability and possesses the greatest challenge in the synthesis. Only the Liu group reported the synthesis of monocyclic 1,3‐azaborine in 2011 (note that the parent 1,3‐dihydro‐1,3‐azaborine remains elusive),^[^ ^9^ ^]^ whereas *π*‐extended conjugated molecules featuring 1,3‐azaborine have still been an unexplored area.

**Figure 2:**
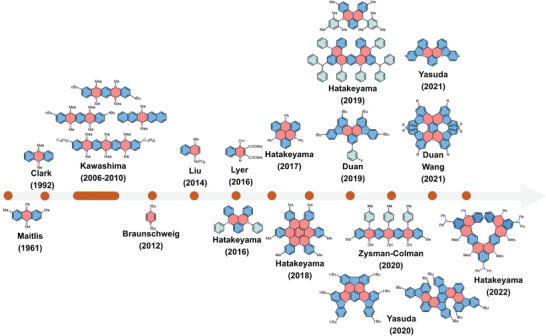
The development history of 1,4‐BN‐heteroarenes showing representative chemical structures.

Despite higher stability of 1,4‐azaborine compared with its 1,3‐isomer, only a few examples were reported before 2006. The first 1,4‐azaborine‐embedded anthracene derivatives were reported by Maitlis and co‐workers in 1961.^[^ ^10^ ^]^ The same backbone with a different substituent was synthesized by Clark and co‐workers in 1992, and the structure was confirmed by single‐crystal X‐ray analysis (**Figure** **2**).^[^ ^11^ ^]^ From 2006 to 2010, Kawashima and co‐workers explored a series of 1,4‐azaborine‐embedded acenes, demonstrating their potential applications in organic light‐emitting diodes (OLEDs) and optical anion sensors.^[^ ^12^ ^]^ Monocyclic 1,4‐azaborine was first reported by Braunschweig and co‐workers in 2012 via rhodium‐catalyzed cycloaddition reaction,^[^ ^13^ ^]^ whereas the parent 1,4‐dihydro‐1,4‐azaborine has remained elusive. In 2014, Liu and co‐workers successfully synthesized 1,4‐azaborine‐embedded naphthalene,^[^ ^14^ ^]^ and disclosed its unique coordination behavior with platinum(II) metal centers. In 2016, Hatakeyama and co‐workers reported 1,4‐azaborine‐based multiresonant thermally activated delayed fluorescence (MR‐TADF) materials and demonstrated their advantages in OLEDs with high external quantum efficiencies (EQEs) as well as excellent color purity.^[^ ^15^ ^]^ Since then, the diversity of 1,4‐BN‐heteroarenes have been greatly enriched and the interest in this class of compounds has been growing rapidly.

There have been more than 60 years since the first synthesis of 1,4‐azaborines, but only recently have they drawn broad attention, especially driven by the development of MR‐TADF materials. Although several reviews partly covering 1,4‐azaborines have been reported in recent years, they are mainly focused on the molecular structures or the specific OLED applications.^[^ ^3c^ ^‐^ ^3^, ^16^ ^]^ Up to date, a chemistry‐oriented review to address the synthetic progresses and challenges, the fundamental structure‐property relationships, and the wide applications (especially beyond MR‐TADF OLEDs) of 1,4‐BN‐heteroarenes has been elusive. Therefore, in this review, we aim to provide a chemistry perspective of BN‐heteroarenes featuring 1,4‐azaborine by summarizing the synthetic strategies and recent advances, as well as their intriguing properties and potential applications in a variety of research fields.

## 2 Synthetic Strategies

According to how the 1,4‐azaborine ring is formed, synthetic strategies toward 1,4‐azaborine derivatives can be generally classified into the following three types: i) transition‐metal‐catalyzed reactions, ii) nucleophilic substitution reactions, and iii) electrophilic C—H borylation reactions. In the following, the three strategies will be discussed in detail together with the synthetic advances.

### 2.1 Transition‐Metal‐Catalyzed Reactions

**Scheme 1:**
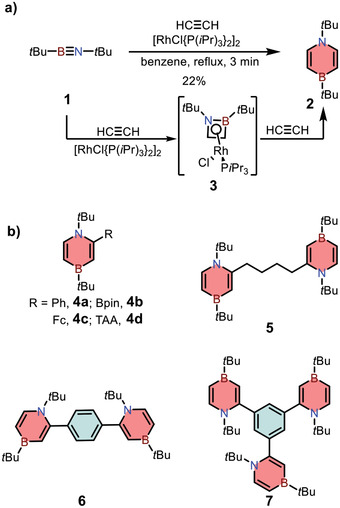
a) Synthetic route to monocyclic 1,4‐azaborine **2** via rhodium‐catalyzed cycloaddition reaction. b) Other 1,4‐azaborine derivatives.

[2+2+2] cycloaddition is a powerful strategy to construct aromatic rings, such as substituted benzene and pyridine derivatives.^[^ ^17^ ^]^ In 2012, Braunschweig and co‐workers tried to prepare 1,2‐azaborines from iminoborane **1** with excess alkyne in the presence of [{(*i*Pr_3_P)_2_RhCl}_2_].^[^ ^13^ ^]^ Surprisingly, they found that the reaction selectively afforded 1,4‐azaborine **2** in moderate yields. The reaction was demonstrated to proceed via intermediate **3** through a tandem [2+2]/[2+4] cycloaddition process, which was different from common alkyne cyclotrimerization reaction (**Scheme** **1**). To further extend the scope of this reaction, in 2016, they synthesized a variety of functionalized 1,4‐azaborines (**4**) with different substituents at C(2) position, including the molecules with two and three discrete 1,4‐azaborine moieties (**5**–**7**).^[^ ^18^ ^]^

**Scheme 2:**
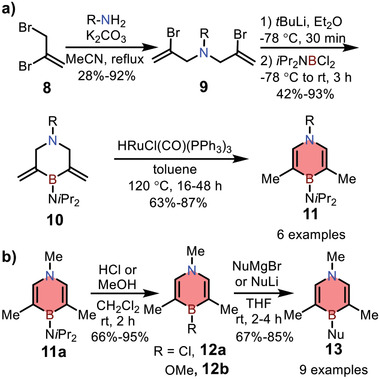
a) Synthetic routes to monocyclic 1,4‐azaborines via Ru‐catalyzed olefin isomerization reaction. b) Nucleophilic substitution towards B‐substituted 1,4‐azaborines.

By comparison, in 2016, Liu and co‐workers reported the synthesis of monocyclic 1,4‐azaborines via a different method.^[^ ^19^ ^]^ As illustrated in **Scheme** **2**, various substituted bis(2‐bromoallyl)amines **9** were used as building blocks for dual Li/Br exchange and the subsequent nucleophilic substitution reactions with *i*Pr_2_NBCl_2_, affording precursor **10** with exocyclic alkenes. Then the corresponding 1,4‐azaborines **11** were obtained efficiently via a Ru‐catalyzed isomerization. Direct replacement of the *N,N*‐diisopropylamino group in **11a** with OMe or Cl group was achieved, and the resulting 1,4‐azaborines with B‐Cl (**12a**) or B‐OMe (**12b**) group could further react with various anionic nucleophiles, leading to functionalized 1,4‐azaborines **13** (Scheme 2). It is worth noting that compounds **102** and **103** were obtained by this approach and exhibited distinct photophysical properties compared with their all‐carbon analogs, which will be discussed in detail in Section 3.1.

**Scheme 3:**
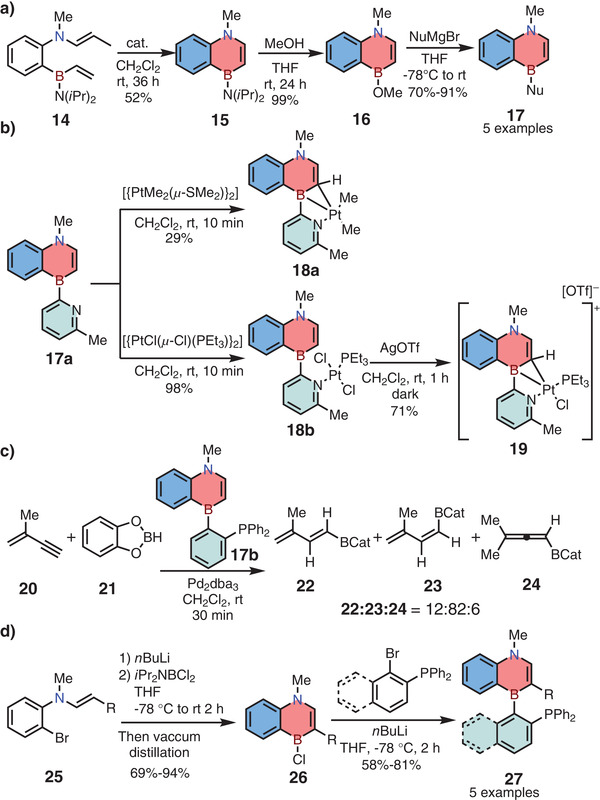
a) Synthetic route to 1,4‐azaborine‐embedded naphthalene via ring‐closing metathesis (RCM) reaction. b) Synthesis of *k* ^2^‐*N*‐*ɳ* ^2^‐BC Pt complex. c) Unique selectivity in Pd‐catalyzed hydroboration reaction. d) Synthesis of 1,4‐azaborine‐embedded SenPhos ligands.

Apart from monocyclic 1,4‐azaborines, Liu and co‐workers also reported the synthesis of 1,4‐azaborine‐embedded naphthalene **15** in 2014.^[^ ^14^ ^]^ The key precursor **14** was achieved from commercially available 2‐bromoaniline in four steps, and then was converted into compound **15** in the presence of Ru‐based Grubbs second‐generation catalyst (**Scheme** **3**a). Similarly, the diisopropylamino substituent in **15** could be quantitatively replaced by methoxy group in the presence of methanol. Then the afforded **16** was further transformed into various functionalized 1,4‐azaborines **17** by nucleophilic substitutions. Interestingly, a unique *k* ^2^‐*N*‐η_2_‐BC mode coordination was found by treating **17a** with [{PtMe_2_(*μ*‐SMe_2_)}_2_] or *trans*‐[{PtCl(*μ*‐Cl)(PEt_3_)}_2_], resulting in charge‐neutral Pt complex **18a** and cationic Pt complex **19**, respectively (Scheme 3b). Notably, the similar complex could not be obtained from the corresponding carbonaceous ligand under the same reaction conditions. Furthermore, the utility of this novel skeleton in organic synthesis was carried out by using 1,4‐azaborine‐based phosphine ligand **17b** for Pd‐catalyzed 1,3‐enyne hydroboration reaction, leading to preferable *trans* addition products (Scheme 3c).

In 2016, the same group developed a new strategy to access 1,4‐azaborine‐based SenPhos ligands **27** (Scheme 3d).^[^ ^20^ ^]^ The key enamine **25** was treated with *n*BuLi firstly, and then the formed lithium reagents were trapped by *i*Pr_2_NBCl_2_, followed by intramolecular electrophilic borylation to give air‐sensitive **26** in one pot. Finally, the target ligands **27** were obtained successfully by the nucleophilic substitution reaction using phosphine‐containing organolithium nucleophiles. Indeed, this strategy superseded the original method in terms of the scale of preparation. Meanwhile, the use of **27** as ligands in 1,3‐enyne hydroboration reactions showed higher *trans* selectivity (see Section 3.3).

**Scheme 4:**
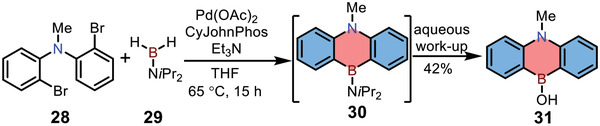
Synthetic route to 1,4‐azaborine‐embedded anthracene by Pd‐catalyzed borylation.

In 2017, Chatani and co‐workers reported the preparation of 1,4‐azaborine‐embedded anthracene by palladium‐catalyzed borylation (**Scheme** **4**).^[^ ^21^ ^]^ Dihalide **28** was selected as the key building block and reacted with diisopropylaminoborane **29** in the presence of Pd(OAc)_2_, forming 1,4‐azaborine **30** with air‐sensitive character in one pot. The intermediate **30** could be further hydrolyzed by MeOH or aqueous NH_4_Cl, affording product **31** in 42% yield. Although the same skeleton of compound **31** was already reported in 1961 by nucleophilic substitution, this is the first example of constructing 1,4‐azaborine‐embedded anthracene without using organolithium reagents. In addition, this palladium‐catalyzed borylation could be explored for the preparation of various dibenzo‐1,4‐heteroborines containing O or S atoms in moderate yields.

### 2.2 Nucleophilic Substitution Reactions

**Scheme 5:**
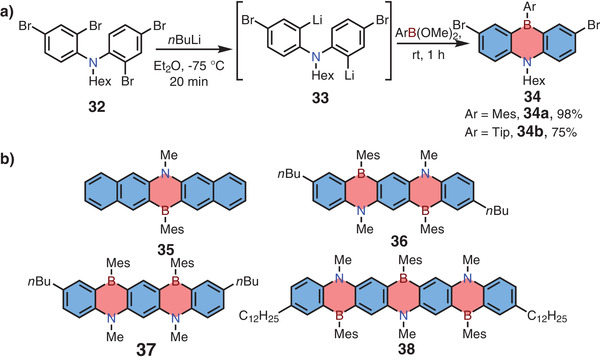
a) Nucleophilic substitution reaction to afford 1,4‐azaborine‐embedded anthracenes and b) 1,4‐azaborine‐embedded pentacene and heptacene.

The first 1,4‐azaborine‐embedded anthracene was synthesized by Maitlis in 1961 via nucleophilic substitution reaction, and the single‐crystal X‐ray structure of the same skeleton was further confirmed by Clark and co‐workers in 1992.^[^ ^10^, ^11^ ^]^ From 2006 to 2010, Kawashima and co‐workers developed a series of 1,4‐azaborine‐based linear acenes by the same synthetic strategy (**Scheme** **5**).^[^ ^12a^ ^–^ ^d^ ^]^ Taking 1,4‐azaborine‐embedded anthracene **34** for example, the synthesis started from Li/Br exchange of 2,4‐dibromo‐*N*‐(2,4‐dibromophenyl)‐*N*‐hexylaniline **32** to form intermediate **33**, followed by nucleophilic substitution reaction with MesB(OMe)_2_ or TipB(OMe)_2_ to give target compounds in one pot. Furthermore, 1,4‐azaborine‐based acenes **35**–**38** were obtained successfully by the same method. Nearly coplanar geometry of compound **36** was determined by X‐ray single‐crystal analysis, indicating good *π*‐extension across the conjugated framework. This character was in accordance with the bathochromic shift of both the absorption and emission maxima of **38** (the maximum *λ*_abs_ at 608 nm and *λ*_em_ at 625 nm) compared with **36** (*λ*_abs_ at 523 nm, *λ*_em_ at 534 nm). Meanwhile, *π*–*π* stacking interaction of compound **36** was hampered by the large steric group (mesityl group, Mes), which was beneficial to the high photoluminescence quantum yield (PLQY) of up to 69%.

**Scheme 6:**
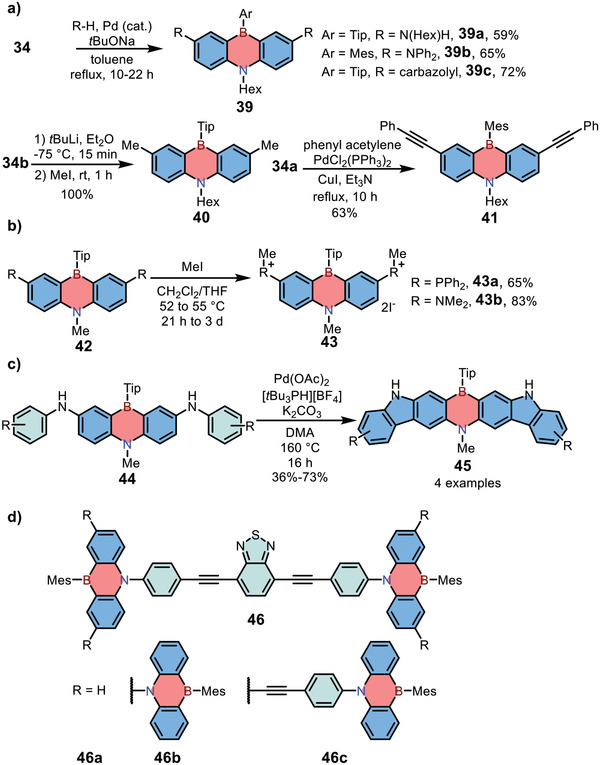
a–d) Synthesis of 1,4‐azaborine‐embedded anthracene derivatives.

Apart from novel skeletons, Kawashima and co‐workers also explored various peripherally functionalized 1,4‐azaborine‐embedded anthracene derivatives from **34** via the Buchwald‐Hartwig amination, Li/Br exchange in conjunction with nucleophilic substitution, or Sonogashira cross‐coupling reactions, giving products **39**, **40,** and **41**, respectively (**Scheme** **6**).^[^ ^12b^ ^]^ Notably, although compound **39c** with carbazolyl moiety shared the similar low‐energy absorption maximum with the parent 1,4‐azaborine‐embedded anthracene (around 400 nm), the former exhibited an ultrahigh PLQY up to 100%. In 2019, the potential of **39c** as emitting materials in OLEDs was examined by Cheng and co‐workers by replacing hexyl group at nitrogen with phenyl moiety, and the resulting device exhibited a narrow emission band (full‐width at half‐maximum, FWHM = 51 nm) with TADF character.^[^ ^22^ ^]^ Kawashima and co‐workers also reported the synthesis of water‐soluble dicationic 1,4‐azaborines **43** by treating precursor **42** with iodomethane and further demonstrated their potential as optical anion sensors (Scheme 6b).^[^ ^12e^ ^]^ In 2021, Agou and co‐workers reported the synthesis of *π*‐extended 1,4‐azaborines **45** via palladium‐catalyzed C—H activation reaction. Through fusing carbazole moieties, compounds **45** showed improved PLQY and higher electrochemical stability.^[^ ^23^ ^]^ On the other hand, Kawashima and co‐workers further studied the functionalization of 1,4‐azaborine‐embedded anthracenes at the nitrogen position and synthesized donor–acceptor type *π*‐conjugated dendrimers **46** successfully.^[^ ^12c^ ^]^ The emission of dendrimers **46a** and **46c** both revealed obviously bathochromic shift (*λ*_em_ > 600 nm) because of the intramolecular charge transfer (ICT) from the azaborine dendrons to the benzothiadiazole core. By contrast, the non‐emissive character of ICT state in **46b** was responsible for the absence of emission around 600 nm.

**Scheme 7:**
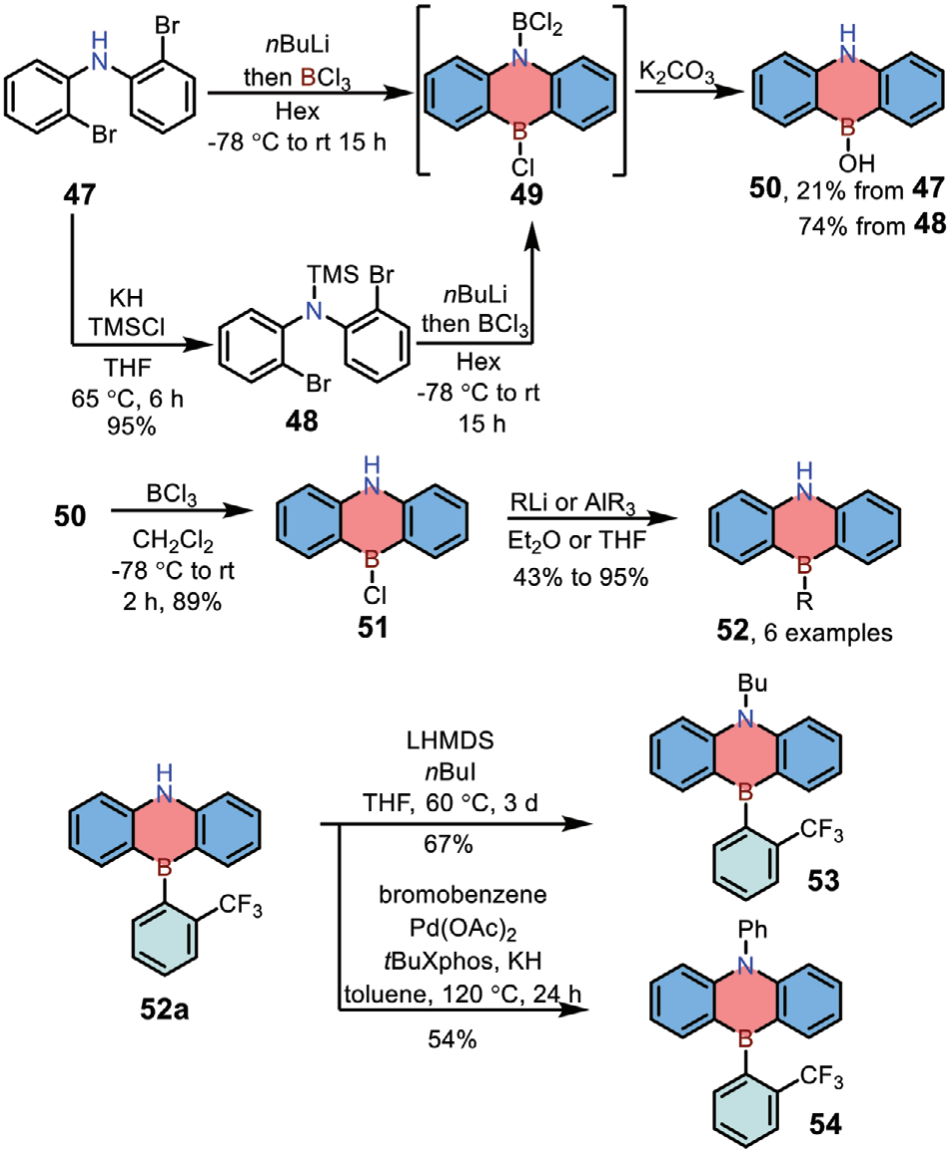
Synthesis of 1,4‐azaborine‐embedded anthracenes via orthogonal functionalization.

In 2019, Yamashita and co‐workers further explored the scope of functionalized 1,4‐azaborine‐embedded anthracenes, achieving compounds **52**–**54** with various substituents at nitrogen or boron positions (**Scheme** **7**).^[^ ^24^ ^]^ Bis(2‐bromophenyl)amine **47** was used as the starting material and reacted with *n*BuLi, then the reaction was trapped by BCl_3_, giving air‐sensitive intermediate **49** with the 1,4‐azaborine backbone. Then compound **49** was further quenched by aqueous K_2_CO_3_, affording corresponding boronic acid **50** in 21% yield for two steps. Notably, compound **50** could also be prepared in 70% yield from **47** via intermediate **48**. Functionalization of **50** on the boron atom was achieved by the activation of hydroxyl group in conjunction with the nucleophilic substitution, giving products **52** in 43–95% yields. Finally, functionalization of **52a** was carried out via nucleophilic alkylation or Buchwald–Hartwig amination, affording target compounds **53** and **54**, respectively.

**Scheme 8:**
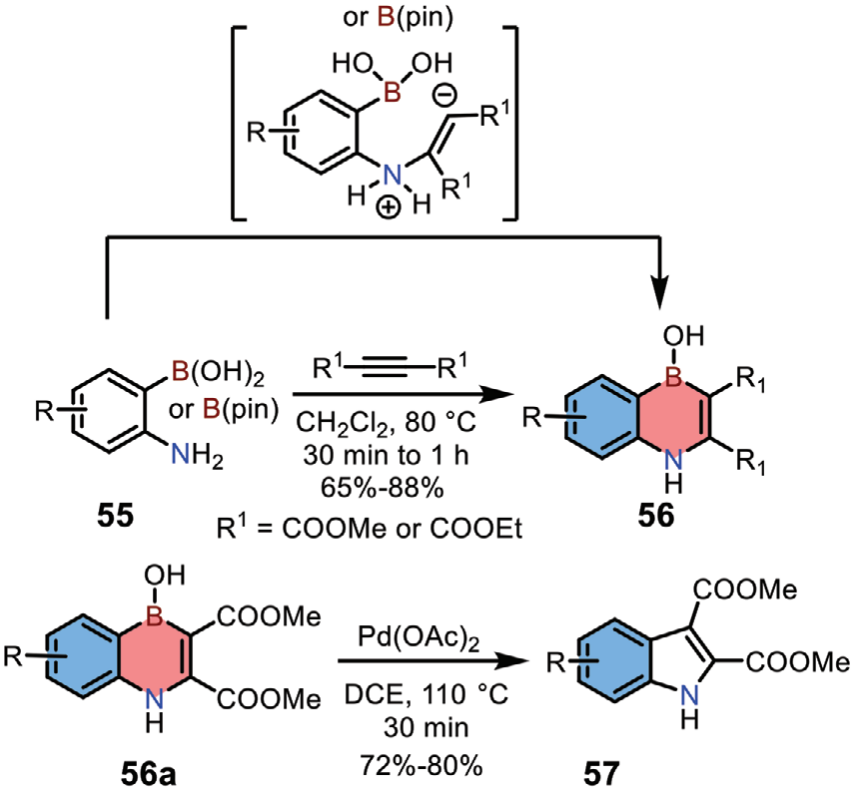
Synthesis and transformation of 1,4‐azaborine‐embedded naphthalenes.

In 2015, Iyer and co‐workers reported the synthesis of 1,4‐azaborine‐embedded naphthalenes **56** by the nucleophilic [4+2] annulation of 2‐aminophenylboronic acids/boronates **55** with alkynes (**Scheme** **8**).^[^ ^25^ ^]^ The scope of this reaction was broad, and various substituted 1,4‐azaborines **56** were afforded in good yields (65–88%). Furthermore, compounds **56a** could be further transformed to corresponding indoles **57** in the presence of palladium acetate.

### 2.3 Electrophilic C—H Borylation Reactions

**Scheme 9:**
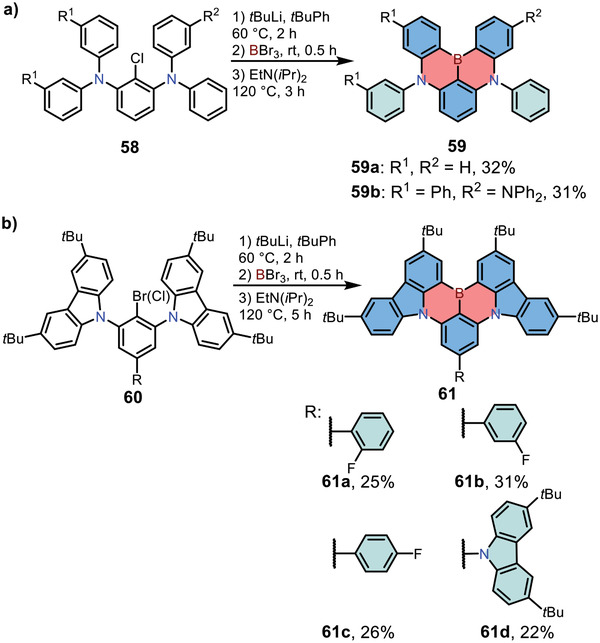
a,b) Preparation of N,B,N‐type 1,4‐azaborine‐based PAHs via electrophilic direct C—H borylation reaction.

Electrophilic direct C—H borylation reaction was first reported in the 1950s, and a great number of conjugated molecules incorporating boron atoms have been explored via this method, including 1,2‐azaborine‐based PAHs.^[^ ^3^, ^26^ ^]^ In 2016, Hatakeyama and co‐workers reported the synthesis of N,B,N‐type 1,4‐azaborines **59** by the electrophilic C—H borylation.^[^ ^15^ ^]^ The obtained compounds revealed separated HOMO–LUMO distribution and exhibited narrow emission bands (FWHM = 28 nm for both two compounds) as the emitting layer in OLED devices. As shown in **Scheme** **9**a, the key precursor **58** were prepared from commercially available 1‐bromo‐2,3‐dichlorobenzene via Buchwald–Hartwig coupling reactions. Then **58** was treated with *t*BuLi for Li/Br exchange, and the intermediate was trapped with BBr_3_ to form one C—B bond. The final two C—B bonds were constructed by intramolecular direct C—H borylation in *tert*‐butylbenzene at high temperature, forming **59** in one pot. To tune the emission properties of 1,4‐azaborines **59**, in 2019, Zhang and co‐workers reported the synthesis of carbazole‐fused 1,4‐azaborines **61** by the similar synthetic strategy (Scheme 9b).^[^ ^27^ ^]^ Precursor **60** was achieved by the nucleophilic substitution reaction of 2‐bromo‐1,3,5‐trifluorobenzene or the nucleophilic substitution in conjunction with Suzuki coupling reactions from 5‐bromo‐2‐chloro‐1,3‐difluorobenzene. Compounds **61a** with electron‐deficient unit at the *para*‐position to boron exhibited a red‐shifted green emission without compromising the color purity due to the enhanced ICT effect.

**Scheme 10:**
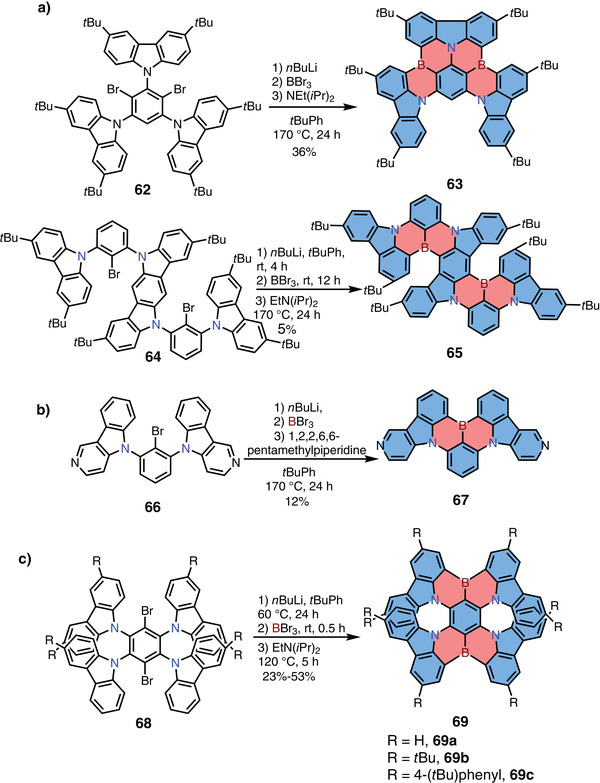
a–c) Synthetic routes to *π*‐extended 1,4‐azaborine derivatives.

Varying the substituents is effective to systematically modulate the emission properties of these N,B,N‐type 1,4‐azaborine‐based MR‐TADF materials, which will be discussed in Section 3.6 (Figure  6) and will not be detailed here. We would, however, like to highlight a series of novel N,B,N‐type 1,4‐azaborine skeletons that were synthesized via the C—H borylation method in recent years (**Scheme** **10**). For example, in 2020, Yasuda and co‐workers reported the synthesis of N,B,N‐type 1,4‐azaborines **63**, **65**, **137**, and **139** from the halogenated precursors that were prepared by the nucleophilic substitution (Scheme 10a and Figure  6).^[^ ^28^ ^]^ The emission maxima of these compounds were well tunable covering nearly the whole visible region (from 466 to 615 nm) by increasing (or decreasing) the electronic strength of boron (as acceptor) and nitrogen (as donor) atoms. In 2021, Yasuda and co‐workers further developed a new family of N,B,N‐type 1,4‐azaborine derivatives **67**, **134**, and **135** by the similar strategy, demonstrating their potential as color‐tunable MR‐TADF materials (Scheme 10b and Figure  6).^[^ ^29^ ^]^

In 2021, Zhang and co‐workers reported the synthesis of N,B,N‐type 1,4‐azaborine‐embedded PAHs **69a,b** (Scheme 10c).^[^ ^30^ ^]^ Precursors **68** were prepared by nucleophilic substitution reaction from 1,4‐dibromo‐2,3,5,6‐tetrafluorobenzene in excellent yields. Then compounds **68** were treated with *n*BuLi for Li/Br exchange, and the lithium reagent was reacted with BBr_3_, followed by intramolecular C—H borylation at high temperatures to form **69a,b** in one pot. The OLED devices employing compounds **69a,b** as the emitters exhibited deep‐red emission with EQEs up to 28%. Notably, at the same time, Wang and co‐workers independently reported compounds **69a,b** as well as compound **69c**, and disclosed their excellent chiroptical properties as a new type of B,N‐embedded double hetero[7]helicenes, such as high absorption dissymmetry factors (*g*_abs_) and efficient circularly polarized luminescence (see Section 3.2).^[^ ^31^ ^]^

**Scheme 11:**
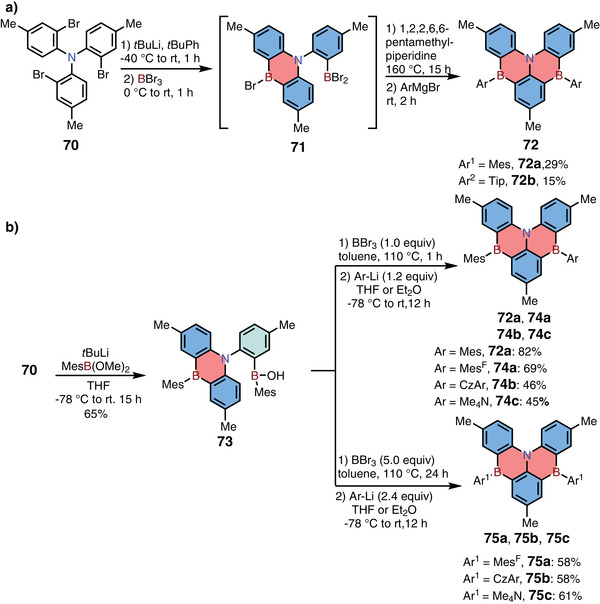
a,b) Syntheses of B,N,B‐type 1,4‐azaborine‐based PAHs.

In addition to the well‐developed N,B,N‐type 1,4‐BN‐heteroarenes, in 2019, Hatakeyama and co‐workers also reported the synthesis of B,N,B‐type 1,4‐azaborine‐based PAHs **72**, which shared the similar backbone with compounds **59** (**Scheme** **11**a).^[^ ^32^ ^]^ In their synthesis, the key precursor **70** was treated with *t*BuLi first, and then the resulting lithium reagent was trapped by two equivalents of BBr_3_, forming intermediate **71** with one 1,4‐azaborine ring. The second 1,4‐azaborine ring was built up via direct electrophilic borylation reaction. Finally, the bromine at boron atoms was replaced by steric groups to improve the stability. Compounds **72** were successfully used as MR‐TADF materials and displayed sky‐blue emission (around 480 nm). Besides, in 2020, Wang and co‐workers reported the same skeleton (**74** and **75**) via a different strategy (Scheme 11b).^[^ ^33^ ^]^ In their synthesis, the same precursor **70** was treated with *t*BuLi firstly, and then reacted with MesB(OMe)_2_, forming air‐stable 1,4‐azaborine **73**. Compound **73** was found to display a unique B‐Mes bond cleavage reactivity under different equivalents of BBr_3_, affording 1,4‐azaborines with various steric groups.

**Scheme 12:**
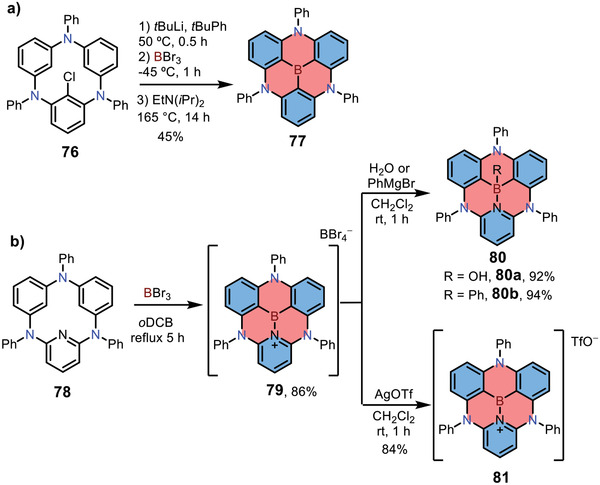
a,b) Synthesis of 1,4‐azaborine‐embedded [3]triangulenes.

Electrophilic C—H borylation can also be used for constructing 1,4‐azaborine‐embedded triangulenes. In 2017, Hatakeyama and co‐workers reported the synthesis of 1,4‐azaborine‐embedded [3]triangulenes **77** (**Scheme** **12**).^[^ ^34^ ^]^ The key precursor triphenylcyclotrianiline **76** was synthesized from 2‐chloro‐diphenylbenzene‐1,3‐diamine via two intermolecular and one intramolecular Buchwald‐Hartwig amination reactions. Then the boron atom was introduced into the central core via nucleophilic substitution in conjunction with electrophilic borylation reaction, forming 1,4‐azaborine **77** with a fully constrained structure. Note that this strategy could be further expanded to construct various hetero[3]triangulenes with different central heteroatoms/groups (e.g., P, P═S, P═O, and SiMe). In 2021, the same group synthesized cationic 1,4‐azaborine **79** with tetrabromoborate (BBr_4_^−^) as a counter ion through the same method (Scheme 12b).^[^ ^35^ ^]^ Due to the electron‐accepting character of boron atom, compound **79** was sensitive towards nucleophilic reagents, which could lead to air‐stable derivatives **80**. Interestingly, treatment of compound **79** with AgOTf afforded another cationic derivative **81**, which showed high air stability in the solid state. Salt **81** exhibited reversible two‐electron reduction waves in electrochemistry and could be further transformed into 2D brickwork‐type *π*‐electronic ion pair by treating it with 1,2,3,4,5‐pentacyanocyclopentadienyl anion, demonstrating its potential as electronic materials.

**Scheme 13:**
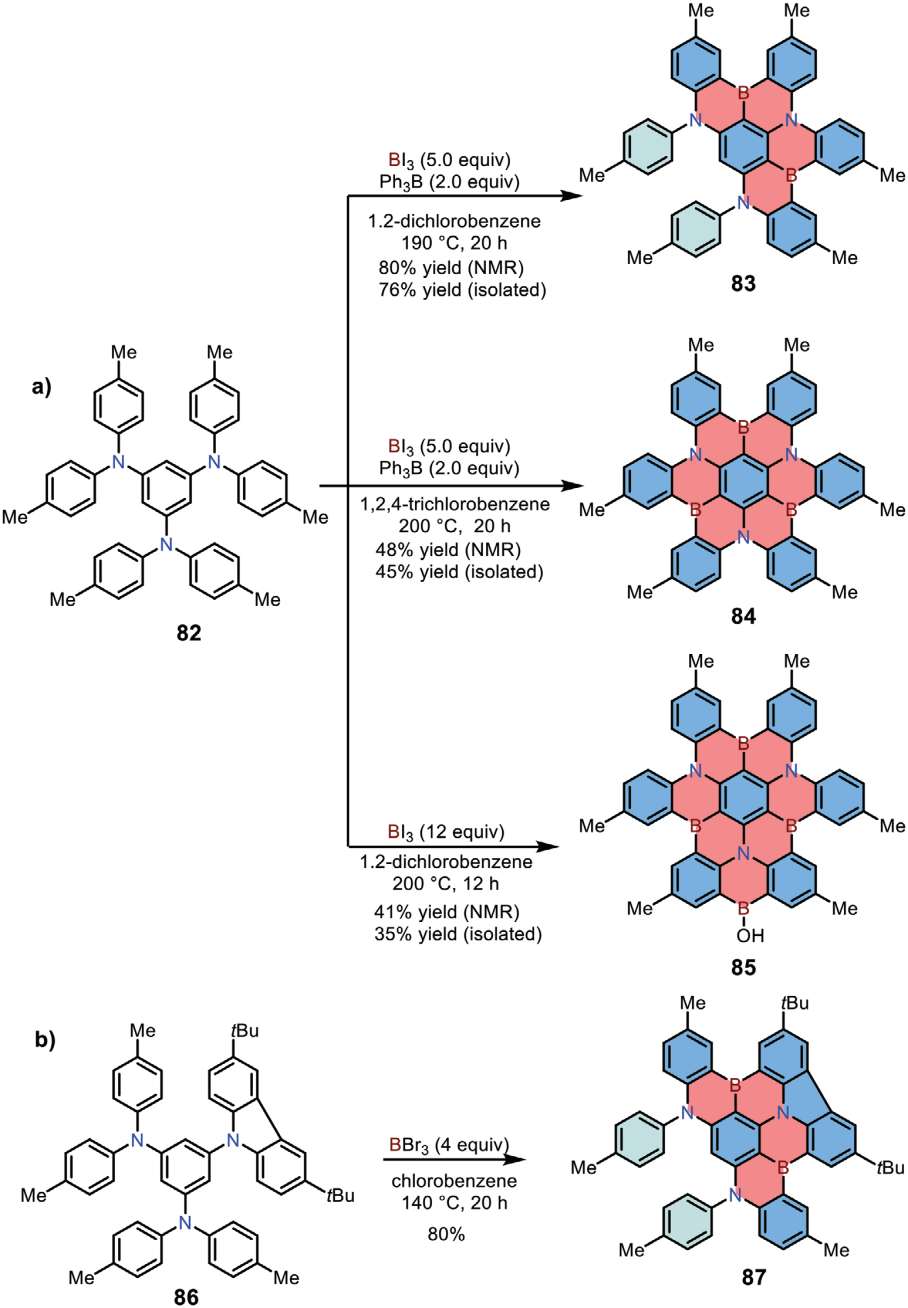
a,b) Syntheses of 1,4‐BN‐heteroarenes via one‐pot multiple electrophilic borylation reactions.

The above examples generally involve two steps for forming new C—B bonds: nucleophilic substitution of corresponding lithium reagent with BBr_3_ and subsequent direct C—H borylation reaction. The procedure is somewhat complicated, so it would be simpler if 1,4‐azaborine‐based PAHs could be obtained by direct C—H borylation on electron‐rich substances. In 2018, Hatakeyama and co‐workers reported the synthesis of multiple 1,4‐azaborine‐embedded PAHs via one‐pot direct borylation method (**Scheme** **13**a).^[^ ^36^ ^]^ The *p*‐phenyl‐1,3,5‐triamine **82** was synthesized from 1,3,5‐tribromobenzene via Buchwald‐Hartwig coupling reaction. The products of direct electrophilic borylation highly depended on the reaction temperature as well as the equivalents of triiodoborane (BI_3_). Doubly boron‐fused 1,4‐azaborine **83** was achieved by treating precursor **82** with five equivalents of BI_3_ in the presence of triphenylborane (BPh_3_) at 190°C, whereas threefold boron‐embedded 1,4‐azaborine **84** was obtained as the main product when the reaction temperature was increased to 200 °C in 1,2,4‐trichlorobenzene. Besides, in the presence of excess BI_3_ (without BPh_3_), multi‐borylation of triamine **82** occurred, affording main product **85** with four boron atoms, whereas other boron sources (i.e., BCl_3_ or BBr_3_) could not give **85** at all. In 2021, Hatakeyama and co‐workers reported carbazole‐fused 1,4‐azaborines **87** via the same strategy (Scheme 13b).^[^ ^37^ ^]^ Two boron atoms were selectively introduced at the *ortho*‐position of the carbazolyl group because of the higher electron‐donating abilities of carbazolyl. The resulting carbazole‐fused compound **87** exhibited slightly bathochromic emission (*λ*_max_: 491 nm) compared with **83** (*λ*_max_: 455 nm).

**Scheme 14:**
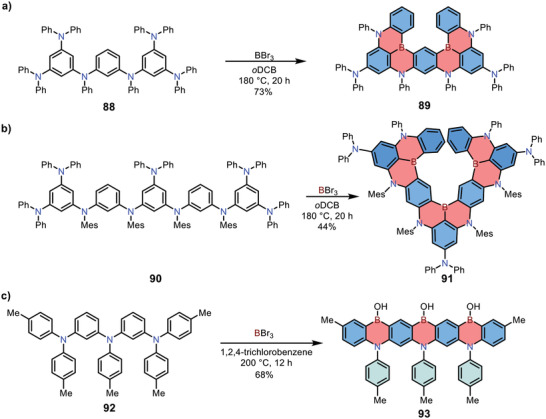
a–c) Synthesis of ladder‐type 1,4‐azaborine‐embedded PAHs via one‐pot multiple electrophilic borylation reactions.

Multiborylation reaction can also be used to build 1,4‐azaborine‐embedded PAHs with other topologies. In 2019, Hatakeyama and co‐workers developed a *π*‐extended 1,4‐azaborine **89** (**Scheme** **14**a).^[^ ^38^ ^]^ The key precursor **88** was synthesized from commercially available 1,3‐dibromo‐5‐chlorobenzene via two sequential Buchwald‐Hartwig coupling reactions. Then **88** was treated with BBr_3_ directly in 1,2‐dichlorobenzene at 180 °C, forming six C—B bonds in one pot to provide **89** in 73% yield. The OLED device employing **89** as the emitter exhibited a narrow emitting band (*λ*_max_: 469 nm, FWHM: 18 nm), demonstrating its advantage as an ultrapure blue‐emitting material. In 2022, the same group reported the synthesis of an expanded 1,4‐azaborine‐based PAH **91** with a twisted structure.^[^ ^39^ ^]^ The product **91** was prepared by treating diphenylamine derivatives **90** with excess BBr_3_ at a high temperature (180 °C), forming nine C—B bonds in one pot (Scheme 14b). The *π*‐extended framework of **91** exhibited narrowband sky‐blue emission with a small FWHM of 16 nm and a high *k*_RISC_ of 4.4 × 10^5^ s^−1^.

In 2020, Zysman‐Colman and co‐workers reported the synthesis of 1,4‐azaborine‐embedded heptacene **93** (Scheme 14c).^[^ ^40^ ^]^ Precursor **92** was obtained through a sequence of Ullman and Buchwald–Hartwig coupling reactions with a moderate yield for two steps. Interestingly, although excess BBr_3_ (18 equivalents) was added, the reaction mainly afforded a structurally rigid linear backbone. Then the reaction was quenched by aqueous sodium acetate and 1,4‐azaborine **93** was achieved successfully. Compound **93** showed a dramatic combination of both MR‐TADF and triplet‐triplet annihilation (TTA) characters and was considered as a highly efficient deep‐blue emitting material for OLEDs.

**Scheme 15:**
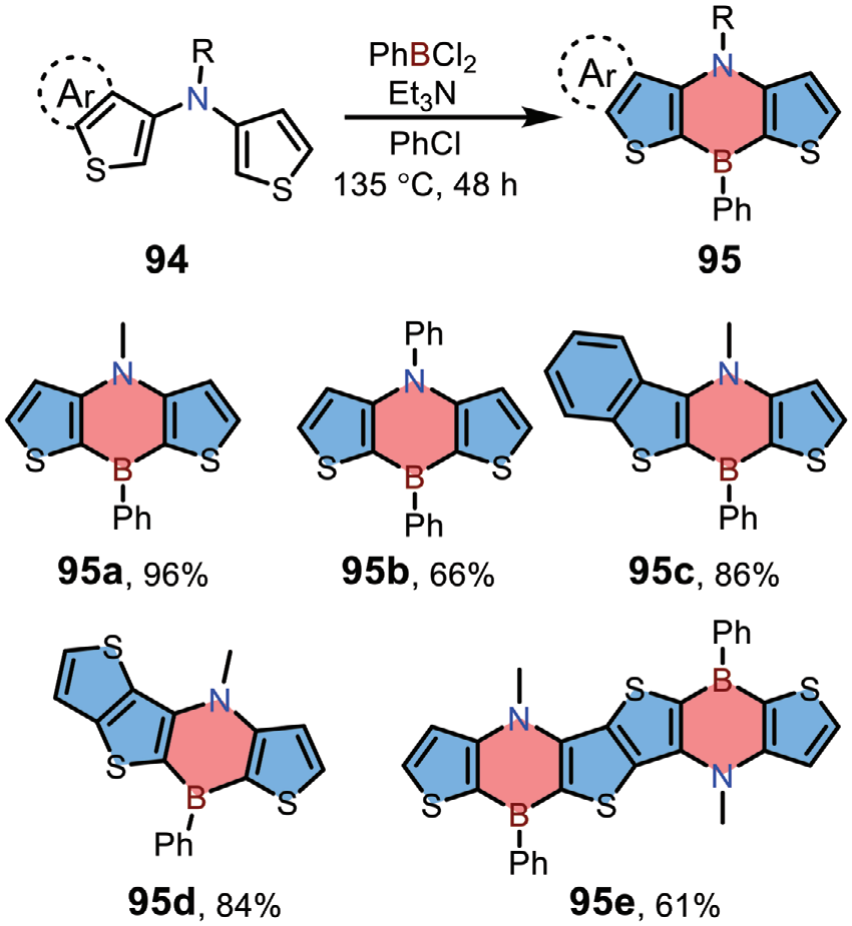
Synthesis of dithieno‐fused 1,4‐azaborines.

In addition to dibenzo‐1,4‐azaborines, Mitsudo and co‐workers also reported the synthesis of dithieno‐fused 1,4‐azaborines **95** (**Scheme** **15**).^[^ ^41^ ^]^ The precursors **94** were prepared from 3‐bromothiophene derivatives via the Buchwald–Hartwig amination, and then were treated with PhBCl_2_ for the direct electrophilic borylations to form two C—B bonds in the presence of triethylamine, giving products **95** in good yields.

**Scheme 16:**
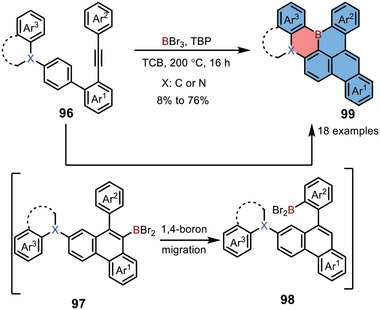
Synthesis of 1,4‐azaborine‐based PAHs via a key 1,4‐boron migration step.

In 2021, Feng and co‐workers developed a series of boron‐embedded PAHs, including 1,4‐azaborines, by a one‐pot cascade reaction (**Scheme** **16**).^[^ ^42^ ^]^ Compounds **96** was selected as the key precursor for electrophilic borylation, and was treated with BBr_3_ in the presence of 2,4,6‐tri‐*tert*‐butylpyridine, forming one benzene ring with a BBr_2_ substituent. Then the obtained intermediate **97** underwent 1,4‐boron migration, affording intermediate **98**. The subsequent intramolecular twofold electrophilic borylations generated a variety of boron‐embedded PAHs, including 1,4‐azaborines **99**. This strategy featured simplicity and a broad substrate scope and could be used for building various expanded B‐doped graphene nanostructures.

## 3 The Value of Incorporating 1,4‐Azaborines

During the past two decades, the chemistry of 1,4‐azaborines has achieved significant development, and numerous compounds have revealed unique properties compared with their carbonaceous counterparts. In this section, we will discuss the intriguing properties and promising applications of 1,4‐azaborine‐based BN‐heteroarenes owing to the unique effect of BN‐incorporation.

### 3.1 Altering the Photophysical Properties

**Figure 3:**
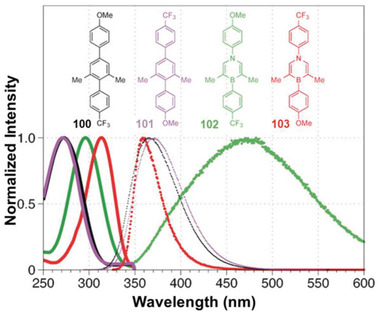
Normalized absorption (solid lines) and emission (dotted traces) spectra of 1,4‐azaborines **102** (green) and **103** (red) and their carbonaceous analogs **100** (black) and **101** (purple) in THF at 10 × 10^‐6^ m. Reproduced with permission.^[^ ^19^ ^]^ Copyright 2016, Wiley‐VCH.

The different *π*‐electron distribution and unique dipole moment of 1,4‐azaborine as compared with benzene endow 1,4‐BN‐heteroarenes with appealing properties. To deeply understand this effect, Liu and co‐workers synthesized two kinds of donor–acceptor‐type 1,4‐azaborines **102** and **103**, manifesting different photophysical properties compared with their isoelectronic counterparts **100** and **101** (**Figure** **3**).^[^ ^19^ ^]^ It was obvious that carbonaceous terphenyls **100** and **101** exhibited similar absorption (*λ*_max_: 272 and 270 nm) as well as emission (*λ*_max_: 365 and 370 nm) bands, which was attributed to the same conjugated backbone. In contrast, compounds **102** and **103** with polar 1,4‐azaborine moiety revealed significant differences in both the absorption and emission spectra. The absorption maximum of **102** was at 296 nm, slightly bathochromic‐shifted compared with 1**00**, but hypochromic shifted than its isomer **103** (*λ*_max_: 313 nm). On the other hand, compound **102** revealed a broad emission band (from 350 to 600 nm) with the maximum at 482 nm, whereas compound **103** exhibited a relatively narrow emission band with the maximum at 359 nm. Meanwhile, due to the electronic effect of the substituents, 1,4‐azaborines **102** and **103** showed significantly different Stokes shifts: the former displayed a relatively large Stokes shift (13037 cm^–1^) compared with the carbon analogs (9367 cm^–1^ for **100** and 10010 cm^–1^ for **101**), while the latter exhibited a dramatically decreased Stokes shift (4094 cm^–1^). Such results demonstrated that **103** has a relatively small structural reorganization between the ground state and the excited state. This example showcased how the incorporation of 1,4‐azaborine greatly affected the photophysical properties of conjugated materials.

### 3.2 Bringing Excellent Chiroptical Properties

**Figure 4:**
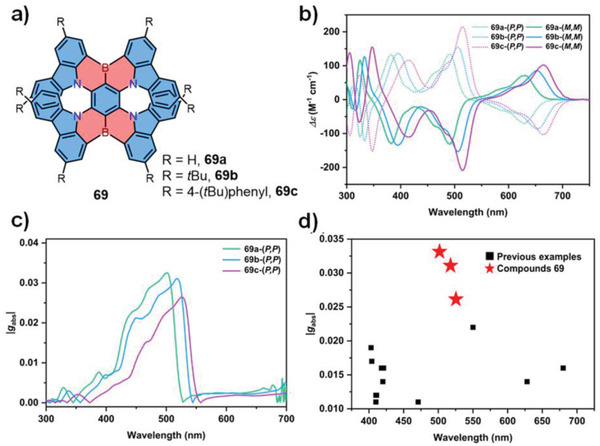
a) Chemical structures of compounds **69a–c**. b) Circular dichroism spectra of **69a**–**c** in dichloromethane solution (1 × 10^–5^ m). c) The corresponding |*g*_abs_| of (*P*,*P*)‐configuration calculated according to the equation *g*_abs_ = Δ*ε*/*ε*. d) Summary of the |*g*_abs_| values of the reported helicenes and compounds **69a–c**. Reproduced with permission.^[^ ^31^ ^]^ Copyright 2021, American Chemical Society.

In recent years, helicene‐based materials have shown great potential in the field of chiral optoelectronic devices, for which significant chiroptical responses in the visible region with high dissymmetry factors are desirable.^[^ ^43^ ^]^ Up to date, only very few helicenes exhibit dissymmetry factors up to 10^−2^ in the visible region, calling for new designs of helicene‐based chiroptical materials. In 2021, Wang and co‐workers reported the synthesis and chiroptical properties of N,B,N‐type 1,4‐azaborine‐embedded double [7]helicenes **69** (**Figure** **4**a).^[^ ^31^ ^]^ The synthetic route to compounds **69** was illustrated in Section 2.3. Compounds **69** exhibited excellent chiral stability with the isomerization barrier of **69a** up to 67.52 kcal mol^‐1^. Each pair of enantiomers of **69a–c** was successfully separated by chiral high‐performance liquid chromatography (HPLC) and their chiroptical properties were characterized. The circular dichroism (CD) spectra of compounds **69a–c** covered the ultraviolet and the whole visible regions (from 300 to 700 nm) with the maximum absorption dissymmetry factors (|*g*_abs_|) reaching 0.033 at 502 nm, representing the highest value for helicene molecules in the visible range (Figure 4b–d).

Theoretical calculations revealed that the transition with the largest |*g*_abs_| was assigned to the S_0_→S_2_ transition (HOMO→LUMO+1). For small organic molecules, the dissymmetry factors can be determined by the simplified equation *g* = 4cos*θ*| ***m*** |/| ***µ*** |. Therefore, larger | ***m*** | and cos*θ* as well as smaller | ***µ*** | can lead to higher *g* values. In compounds **69**, the well‐separated molecular orbital distributions between HOMO and LUMO+1 were observed owing to the opposite resonance effect of boron and nitrogen in the 1,4‐azaborine cores, resulting in lower electric transition dipole moments | ***µ*** | for the S_0_→S_2_ transition. Meanwhile, the |cos*θ*| equaled 1 for all cases. As a result, the incorporation of 1,4‐azaborine core in helicenes **69** was helpful for their high |*g*_abs_|. In addition, strong intramolecular donor–acceptor interactions from the B and N atoms extended the absorption of light to the red region. Furthermore, compounds **69** displayed PLQYs up to 100%, and exhibited tunable circularly polarized luminescence (CPL) responses in the red/NIR region with the CPL brightness up to 40.0 M^–1^ cm^–1^, indicating the great potential of **69** as chiral optoelectronic materials. Given the excellent CPL performance of compounds **69** and their MR‐TADF characters, they might also function as promising candidates for CP‐OLED applications.

### 3.3 Applications As Novel Ligands for Metal Catalysis

**Scheme 17:**
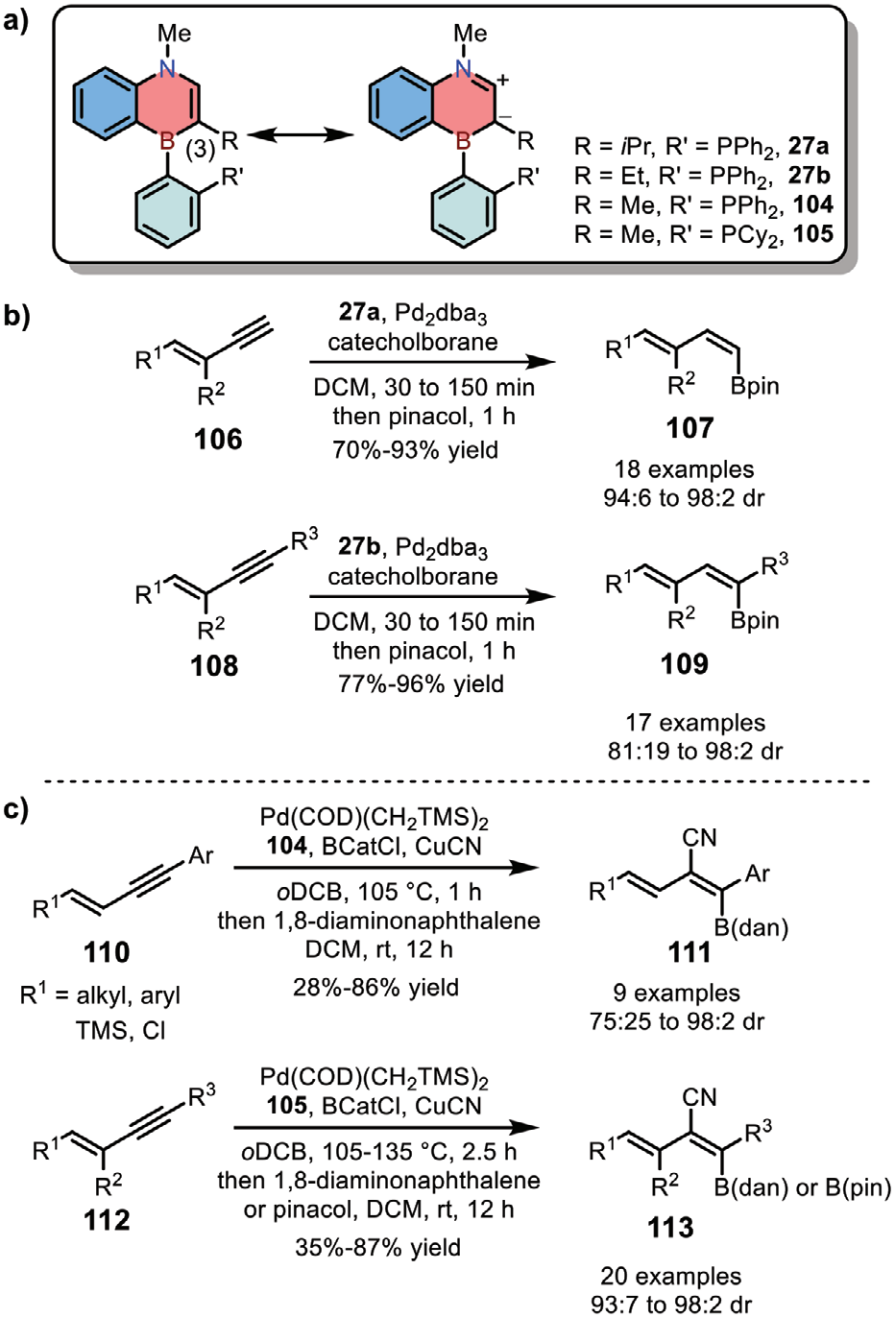
a) Chemical structures of 1,4‐azaborine‐based phosphine ligands. b) Pd‐catalyzed *trans*‐hydroboration and c) *trans*‐cyanoboration of 1,3‐enynes.

In 2014, Liu and co‐workers reported the synthesis of 1,4‐azaborine‐embedded naphthalenes **17** (see Section 2.1).^[^ ^14^ ^]^ Theoretical calculations revealed that the 1,4‐azaborine ring showed a substantial iminium resonance contribution, resulting in a significant donating feature of the C(3) atom (**Scheme** **17**a). Furthermore, the electron‐rich C(3) atom together with the neighboring B atom promoted the formation of η_2_‐L‐type complex with Group 10 transition metals (e.g., Pt and Pd). The 1,4‐azaborine‐based complex stimulated the development of 1,4‐azaborine‐based phosphine ligand **17b** for palladium‐catalyzed hydroboration of 1,3‐enyne, leading to preferable *trans* addition products. Given this preliminary result, Liu and co‐workers further investigated the selectivity of Pd‐catalyzed *trans*‐hydroboration reaction by using 1,4‐azaborines **27a** and **27b** as ligands (see Section 2.1 and Scheme 17b).^[^ ^20a^ ^]^ Under optimized conditions, 1,4‐azaborine **27a** was found suitable for the hydroboration of terminal *E*‐1,3‐enyne with various electronically and sterically different substituents, affording *trans*‐products with high yields (70–93%) and selectivity (94:6 to 98:2 dr). On the other hand, hydroboration of internal 1,3‐enyne remained a great challenge for common metal catalysts because that they required the presence of the terminal alkyne proton to operate the catalytic cycle.^[^ ^44^ ^]^ Excitingly, highly efficient internal 1,3‐enyne hydroboration was achieved by using 1,4‐azaborine **27b**. Generally, higher *trans*‐hydroboration selectivity was observed when **R^1^** were aryl groups (86:14 to 98:2 dr) compared with alkyl groups (81:19 to 97:3 dr). Furthermore, small steric hindrance of **R** ^**3**^ was favorable for high *trans*‐hydroboration selectivity. Note that this reaction could be scaled up to gram scale without erosion of stereoselectivity. In addition to hydroboration reaction, Liu and co‐workers also developed two 1,4‐azaborine‐based Senphos ligands **104** and **105**, which displayed tremendous advantages in Pd‐catalyzed *trans*‐selective cyanoboration of 1,3‐enynes (Scheme **17c**).^[^ ^20b^ ^]^ In general, 1,4‐azaborines as ligands were favorable to form *trans*‐configuration in palladium‐catalyzed hydroboration and cyanoboration reactions, demonstrating the essence of developing 1,4‐azaborine‐based ligands for organic catalysis.

### 3.4 Precursors for Atomically Precise BN‐Doped Graphene Nanoribbons

**Scheme 18:**
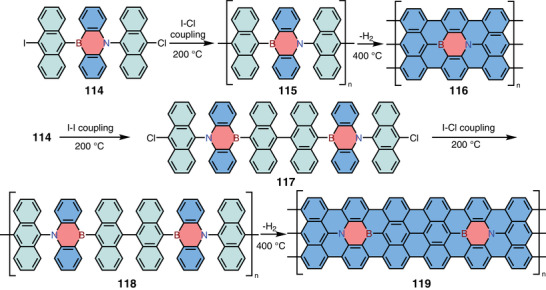
On‐surface synthesis of 1,4‐azaborine‐embedded GNRs.

Graphene nanoribbons (GNRs) are 1D graphene cutouts and have gained wide attention due to their potential applications as the next generation semiconductors.^[^ ^45^ ^]^ Although BN‐doped graphene has been widely explored for optoelectronics and energy‐related applications, the synthesis of site‐specific BN‐doped GNRs with well‐defined structures have been a great challenge. In 2018, Kawai and co‐workers reported the bottom‐up synthesis of 1,4‐azaborine‐embedded armchair GNRs **116** and **119** (**Scheme** **18**).^[^ ^46^ ^]^ Precursor **114** was designed and synthesized from *N*‐(2‐bromophenyl)‐10‐chloro‐*N*‐phenylanthracen‐9‐amine. After deposition onto a Au(111) surface, the monomer underwent polymerization upon heating (200 °C) and then cyclodehydrogenation at a higher temperature (400 °C) to give GNRs. Because of the asymmetric character of the 1,4‐azaborine core, two different segments of GNRs were formed successfully via two different reaction paths. According to theoretical calculations, the GNRs lay on the gold surface with the relative adsorption height of −2 pm for boron and +4 pm for nitrogen. Therefore, the elemental character of 1,4‐azaborine cores was directly resolved by non‐contact atomic force microscopy (nc‐AFM). Such an elemental difference was correlated to the van der Waals radii as well as the local electron density. This elemental‐sensitive measurement marked an important step in the analysis of functionalized 2D carbon materials. This work indicated that sophisticated BN‐doped graphene nanostructures could be obtained through the bottom‐up precision synthesis by using rationally designed BN‐heteroarene precursors.

### 3.5 Severing As Conducting Materials in Single‐Molecule Devices

**Figure 5:**
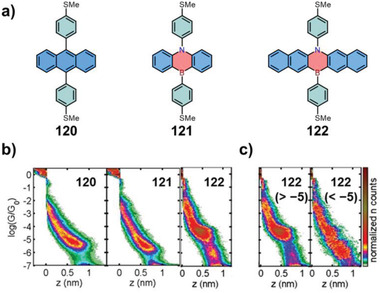
a) Chemical structures of compounds **120–122**. b) 2D histograms for compounds **120–122**. c) Independent 2D histograms of two separated groups for **122**: traces without and with plateaus below log(*G*/*G*_0_) = −5, respectively. Reproduced with permission.^[^ ^50^ ^]^ Copyright 2021, Wiley‐VCH.

Since the first single‐molecule conductance measurement by Reed and co‐workers in 1997,^[^ ^47^ ^]^ a great number of *π*‐conjugated organic molecules have been employed as functional components for the development of single‐molecule devices.^[^ ^48^ ^]^ Among them, heteroatom‐containing molecules have attracted wide attention due to their unique electronic structures as well as tunable electron transport properties.^[^ ^49^ ^]^ However, there are rare examples that the electrons pass directly through the heterocycles. In 2021, González and co‐workers selected 1,4‐azaborines **121–122** as models, and studied their single‐molecule charge transport properties (**Figure** **5**a).^[^ ^50^ ^]^ The 1,4‐azaborine core was embedded in the center of the skeleton, and the linking groups were strategically located at the 1,4‐azaborine core, ensuring that the electron went through the fully conjugated 1,4‐azaborine ring.

The synthesis of compounds **121** and **122** was achieved via the nucleophilic substitution method (Section 2.2). The single‐molecule conductance (*G*) measurements of 1,4‐azaborine **121** and its carbon analog **120** were performed firstly for understanding the effect of 1,4‐azaborine ring on the electron transport properties (Figure 5b). Both compounds exhibited a similar shape of conductance cloud with a broad and sloping conductance signal located between log(*G*/*G*_0_) = −5 and −6. Meanwhile, a slight shift towards lower values was observed for **121**. The Gaussian fitting of the conductance peaks revealed that the log(*G*/*G*_0_) values were −5.3 ± 0.31 for **120** and −5.6 ± 0.33 for **121**, respectively. The similar log(*G*/*G*_0_) value of these two compounds demonstrated that the 1,4‐azaborine core not only permitted the electron transport through the skeleton, but also resulted in a minimum decrease of the conductance compared with that of the all‐carbon structure. Therefore, 1,4‐azaborines could be used as alternatives for investigating the single‐molecule conductance of their air‐sensitive carbon analogs. Then they further tested the single‐molecule conductance of the 1,4‐azaborine‐based pentacene **122**. The 2D histogram of **122** displayed a well‐defined flat cloud of high conductance (80% of the total traces) at around log(*G*/*G*_0_) = −4.5 as well as a less clear conductance cloud (20% of the total traces) below log(*G*/*G*_0_) = −5. Nevertheless, a linker‐to‐linker configuration of the high conductance for **122** was excluded because of the short plateau length. Furthermore, the independent 2D histograms of two separated groups showed that the direct interaction of the electrodes with the central 1,4‐azaborine‐based pentacene unit was responsible for the observed high‐conductance plateaus for **122** (Figure 5c). Finally, the typical conductance of **122** was determined to be log(*G*/*G*_0_) = −5.9 ± 0.40, comparable to compound **121**, indicating that the lateral extension of the central 1,4‐azaborine had little effect on the conductance. This work provided a novel strategy for investigating the single‐molecule conductance of larger acenes by incorporating 1,4‐azaborine moieties.

### 3.6 Applications in MR‐TADF OLEDs

**Figure 6:**
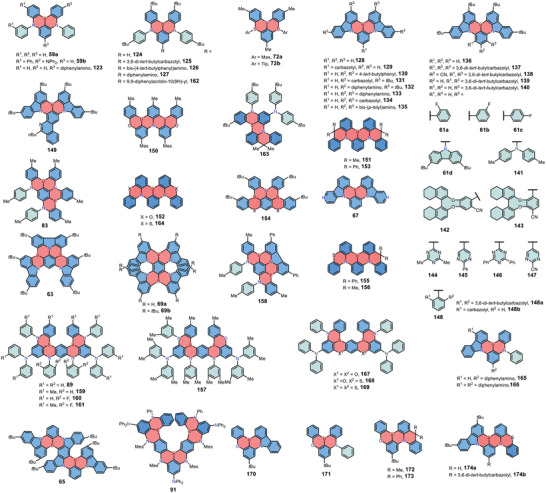
Chemical structures of recently reported MR‐TADF emitters employed for OLED devices.

**Figure 7:**
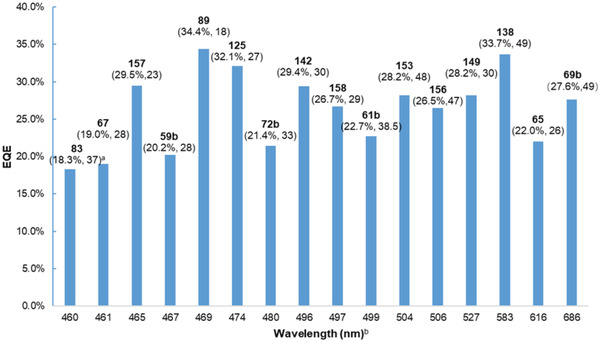
Representative device performances of previously reported MR‐TADF compounds. a) Maximum EQE and FWHM (nm) of the electroluminescence; b) Maximum electroluminescence wavelength.

Recently, 1,4‐azaborine‐based PAHs have gained enormous attention owing to their intriguing advantages in MR‐TADF OLEDs. Due to the opposite resonance effect of boron and nitrogen atoms, 1,4‐azaborines can significantly separate HOMO and LUMO at different positions without introducing donor (D) or acceptor (A) groups. Compared with traditional DA‐type TADF systems, MR‐TADF materials usually reveal small reorganization energy to facilitate high radiative decay rates and low vibration frequency to suppress the nonradiative transitions, and thus exhibit stronger absorption oscillator strengths, smaller FWHMs, and higher PLQYs.^[^ ^3e^ ^]^ In this section, we do not aim at a comprehensive summary of the relevant advance, as there have been several reviews focusing on MR‐TADF emitters.^[^ ^2^, ^3^ ^–^ ^e^ ^]^ We hope to provide an overview of the reported structures as well as the device performance to shed light on the structure–property relationships of 1,4‐BN‐heteroarenes for high‐performance OLEDs. The structures that have been successfully employed for MR‐TADF OLED devices are summarized in **Figure** **6**. The performances of representative OLED devices employing MR‐TADF emitters are summarized in **Figure** **7**.

In 2016, Hatakeyama and co‐workers reported the first N,B,N‐type 1,4‐azaborine‐based MR‐TADF materials **59a** and **59b**.^[^ ^15^ ^]^ OLED devices based on these two compounds both exhibited ultrapure blue emission (*λ*_max_: 467 nm, FWHM: 28 nm for both two compounds) with high EQEs up to 20.2%. Although the device still suffered from serious efficiency roll‐off, this novel molecular design strategy offered an effective way to realize narrowband emission with promising device performance. To improve the EQE of N,B,N‐type 1,4‐azaborines as blue emitters, in 2018, Huang and co‐workers developed a carbazolyl‐substituted 1,4‐azaborine **125**.^[^ ^51^ ^]^ According to theoretical calculations, the introduction of peripheral carbazolyl group could effectively enhance MR‐effect and promote the radiative decay efficiency, and thus resulted in **125** with low efficiency roll‐off, an ultrahigh EQE (32.1%), and a small FWHM (27 nm). Furthermore, the potential of **125** as a host material for a yellow phosphorescent emitter was also demonstrated, and the device showed a significantly reduced turn‐on voltage of 3.2 V and a high EQE of 22.2%. Up to date, a variety of electron‐rich (or electron‐withdrawing) groups have been introduced onto this conjugated skeleton to optimize the device performance (Figure 6).^[^ ^37^, ^52^ ^]^

Interestingly, in this V‐shaped skeleton, the number and positions of boron as well as nitrogen atoms showed limited effect on the emission wavelength. For example, in 2019, Hatakeyama and co‐workers reported B,N,B‐type 1,4‐azaborines **72** and investigated their potential as MR‐TADF materials.^[^ ^32^ ^]^ Although compounds **72** were composed of two electron‐withdrawing boron atoms and only one electron‐rich nitrogen compared with the previously reported 1,4‐azaborines **59**, only a slightly bathochromic shift in the emission spectra was observed with the maximum at 481 nm and 480 nm for **72a** and **72b**, respectively.

In order to achieve long‐wavelength emission of MR‐TADF materials, in 2019, Zhang and co‐workers developed a new carbazole‐fused backbone **61a** and studied the influence of peripheral units, achieving narrowband green emission successfully.^[^ ^27^ ^]^ Based on theoretical calculations, they found that a more delocalized HOMO/LUMO distribution and a slightly reduced energy gap (from 3.66 to 3.42 eV) were observed by replacing diphenylamine moieties in **59** with carbazole units. The bathochromic shift was further boosted by introducing electron‐withdrawing groups at the *para* position of boron, giving compounds **61a** with an enhanced ICT character and reduced energy gaps (around 3.30 eV) for green emission without compromising the color fidelity, whereas the electron‐rich substituent displayed very limited modulating effect. 1,4‐BN‐heteroarenes **61a** represented the first narrowband green MR‐TADF materials with the emission peaks of 493–501 nm, FWHMs of 32–40 nm and high EQEs up to 22.7%. In recent years, a series of carbazole‐fused 1,4‐azaborines were developed following the similar strategy, showing good color purity and device performance (Figure  6).^[^ ^28^, ^29^, ^37^, ^53^ ^]^ In 2020, Duan and co‐workers fused aza‐aromatics onto the 1,4‐azaborine framework and reported a pure‐green MR‐TADF material **149**.^[^ ^54^ ^]^ The target 1,4‐azaborine **149** showed nearly pure‐green emission with a relatively sharp peak at 522 nm, an FWHM of 28 nm, and an extremely high PLQY of 99.7%. The OLED devices employing **149** as the emitter exhibited a maximum EQE and power efficiency of 28.2% and 121.7 lm W^−1^, respectively, with an FWHM of merely 30 nm and Commission Internationale de l'Eclairage (CIE) coordinate *y* of 0.69.

In 2021, Li and co‐workers introduced chiral (*R/S*)‐octahydro‐binaphthol ((*R/S*)‐OBN) units into the carbazole‐fused 1,4‐BN‐heteroarene backbone and reported the first green MR‐TADF materials **142** and **143** with CPL signals.^[^ ^53i^ ^]^ CP‐OLEDs based on **142** and **143** exhibited green emissions with the maximum at 496 and 508 nm, narrow FWHMs of 30 nm and 33 nm, excellent EL performances with EQEs of 29.4% and 24.5%, and EL dissymmetry factors (*g*_EL_) of +1.43 × 10^−3^/−1.27 × 10^−3^ and +4.60 × 10^−4^/−4.76 × 10^−4^, respectively.

In addition to incorporating pendent groups onto the 1,4‐BN‐heteroarene core, expanding the conjugated plane of the rigid framework has also been demonstrated as a useful strategy to modulate their luminescent properties. In 2018, Hatakeyama and co‐workers synthesized three kinds of multiple 1,4‐azaborine‐embedded nanographenes **83**–**85** and demonstrated the potential of **83** as a blue MR‐TADF emitter (*λ*_max_: 460 nm, FWHM: 37 nm, EQE: 18.3%).^[^ ^36^ ^]^ In 2021, they also reported a series of carbazole‐fused 1,4‐azaborines including **141** and **158** via the same strategy (Scheme 13b).^[^ ^37^ ^]^ The OLED devices employing **141** and **158** as emitters exhibited narrowband sky‐blue (*λ*_max_: 477 nm, FWHM: 27 nm) and green (*λ*_max_: 497 nm, FWHM: 29 nm) emissions with high EQEs of 21.8% and 26.7%, respectively.

In 2019, Hatakeyama and co‐workers developed a *π*‐extended linear MR‐TADF material **89**.^[^ ^38^ ^]^ The vibronic coupling of **89** between the ground state (S_0_) and the singlet excited state (S_1_) as well as the energy gap between the S_1_ and the triplet excited state (T_1_) was minimized via the MR effect. OLED devices exhibited deep‐blue emission (*λ*_max_: 469 nm) with a FWHM of only 18 nm and an ultra‐high EQE up to 34.4%. In 2022, they further developed an expanded structure **91** based on the similar strategy.^[^ ^39^ ^]^ The resonating *π*‐extension of **91** minimized the singlet–triplet energy gap and enabled rapid reverse intersystem crossing (RISC) with a rate constant (*k*_RISC_) of 4.4 × 10^5^ s^−1^. The solution‐processed OLED devices employing **91** as an emitter exhibited intense sky‐blue emission peaking at 480 nm with an FWHM of 27 nm and a high EQE of 22.9%. In the same year, Kwon and co‐workers reported 1,4‐azaborines **159–161** by incorporating methyl groups or fluorine atoms on the framework of **89**.^[^ ^55^ ^]^ OLED devices employing **161** as the emitter achieved a high EQE up to 33.7%, CIE coordinates of (0.13, 0.06), and a narrow FWHM of 18 nm.

In 2021, Zhang and co‐workers reported 1,4‐BN‐heteroarenes **69a** and **69b** as the first deep‐red MR‐TADF emitters.^[^ ^30^ ^]^ The 1,4‐azaborine cores were introduced to fundamentally overcome the luminescent boundary set by the “energy gap law”. The nonradiative transitions were greatly eliminated by the shallow potential energy surfaces due to the MR effect. The resulting heteroarenes **69a** and **69b** exhibited highly efficient deep‐red emission with PLQYs up to 100% and the maximum at 664 and 686 nm, respectively. OLED devices based on **69a** and **69b** showed small FWHMs of 48 nm and 49 nm and unprecedented high EQEs of 28.1% and 27.6%, respectively.

Recently, embedding 1,4‐azaborine cores in PAHs together with other heteroatoms (e.g., oxygen or sulfur) was found effective to modulate their emission properties.^[^ ^56^ ^]^ In 2020, Hatakeyama and co‐workers designed a *π*‐extended pure‐green MR‐TADF material **150** by fusing a 1,4‐B,O six‐membered ring on the well‐studied 1,4‐azaborine core.^[^ ^57^ ^]^ Furthermore, to realize solution‐processed OLED devices, two novel conjugated polymers with similar ionization potentials (5.61 and 5.77 eV) were also synthesized to facilitate charge recombination. The resulting devices fabricated with **150** showed green emission with the maximum at 506 nm, the CIE coordinate of (0.12, 0.63), an FWHM of 33 nm, and a high EQE up to 21.8%.

In 2021, Kido and co‐workers developed two green MR‐TADF materials **151** and **152** by inserting sp^3^‐carbon or oxygen into the skeleton.^[^ ^58^ ^]^ The helical configuration of these two compounds could effectively suppress the aggregation‐induced fluorescence quenching, resulting in OLED devices with high EQEs of 20.3% and 23.3%, and FWHMs of 49 and 47 nm, respectively. Notably, MR‐TADF materials **151** and **153** with sp^3^‐carbon bridge were also reported by Yang and co‐workers independently in the same year.^[^ ^59^ ^]^ The nonplanar framework in these two compounds significantly boosted the RISC process, resulting in superior device performances (EQEs up to 28.2%) without utilizing any sensitizer. Furthermore, the introduction of bulkier bis(acridan)phenylene groups in **153** were proved effective to boost the efficiency and alleviate roll‐off behavior (1.8% at 100 cd m^−2^).

In 2021, Yasuda and co‐workers developed MR‐TADF material **154** with 1,4‐B,S six‐membered rings showing sky‐blue emission (*λ*_max_: 478 nm, FWHM: 25 nm).^[^ ^60^ ^]^ Through incorporation of two sulfur atoms, the spin–orbit coupling was effectively enhanced, which facilitated the spin‐flipping process between the excited triplet and singlet states, resulting in EQEs up to 21.0 %.

In 2021, Hatakeyama and co‐workers proposed an ultrapure deep‐blue MR‐TADF material **157** by replacing one nitrogen atom in **89** with an oxygen atom.^[^ ^61^ ^]^ As compared with **89**, OLED devices based on **157** exhibited a hypochromic‐shifted emission maximum at 465 nm, an increased FWHM of 23 nm, a decreased EQE of 29.5 %, a lower efficiency roll‐off (0.4%, 0.7%, and 2.6% at 10, 100, and 1000 cd m^−2^, respectively), and about 10 times longer device lifetime (LT_50_ = 314 and 31 h at 100 cd m^−2^,) because of the restricted *π*‐conjugation effect. In the same year, Yasuda and co‐workers introduced chalcogen (oxygen or sulfur) atoms into the same skeleton of **89** and developed three kinds 1,4‐azaborine‐based MR‐TADF emitters **167**, **168,** and **169**.^[^ ^62^ ^]^ Photophysical characterizations in conjunction with theoretical calculations demonstrated that introducing chalcogen (oxygen and sulfur) atoms can finely modulate the emission color while maintaining the narrow bandwidth (FWHM: 18–23 nm). The OLED devices based on these materials exhibited ultrapure blue EL emissions (*λ*_max_:445 to 463 nm) with CIE *y* coordinates in the range 0.04 to 0.08. Furthermore, the ultra‐fast RISC rate (*k*_RISC_ ≈ 10^6^ s^−1^) was observed in the sulfur‐doped compounds **168** and **169**, which contributed to the high EQEs up to 26.9% and 26.8%, respectively, as well as the small efficiency roll‐offs.

In 2022, Lu and co‐workers incorporated one sulfur atom into the well‐studied backbone **136** and reported the asymmetric MR‐TADF material **174**.^[^ ^63^ ^]^ According to theoretical calculations, the sulfur atom in **174** can effectively enhance spin–orbit coupling (SOC) via the heavy atom effect, leading to fast *k*_RISC_ over 1.0 × 10^5^ s^−1^. The OLED devices employing **174** as emitters exhibited pure green emission (*λ*_max_: 516 nm) with high EQEs up to 32.8% without utilizing any sensitizer. Moreover, the devices sufficiently maintained high EQEs of over 23.5% under 1000 cd m^−2^, representing the highest value for all reported green MR‐TADF materials at the same luminescence.

In addition to the planar MR‐TADF materials, in 2021, Chou and co‐workers reported the helical MR‐TADF materials **155** and **156** for CP‐OLEDs. The asymmetric skeleton was composed of one sp^3^‐carbon bridge and a 1,4‐SN six‐membered ring.^[^ ^64^ ^]^ Theoretical calculations revealed that the chiral moiety of two compounds directly participated in the frontier molecular orbitals (FMOs). The enantiomers of **155** and **156** were separated and employed in CP‐OLED devices, which displayed narrow FWHMs of 49/50 and 48/47 nm, high EQEs of 20.6%/19.0% and 22.0%/26.5%, and *g*_EL_ of +3.7 × 10^−3^/−3.1 × 10^−3^ and +1.9 × 10^−3^/−1.6 × 10^−3^, respectively.

In recent years, modification of the 1,4‐azaborine core and the peripheries also provided opportunities to tune the emission color of MR‐TADF materials in a wide wavelength region. In 2020, Yasuda and co‐workers reported a series of 1,4‐azaborine‐based MR‐TADF materials **63**, **65**, **137**, and **139**.^[^ ^28^ ^]^ These compounds allowed the systematic color‐tuning with narrowband emissions (FWHM: 26–54 nm) to cover the entire visible range, ranging from deep‐blue (**63**, *λ*_max_: 469 nm) to green (**137**, *λ*_max_: 515 nm) and to red (**65**, *λ*_max_: 616 nm). OLED devices employing these compounds as emitters exhibited high EQEs of 29.3% for **63**, 31.8% for **137**, 29.3% for **139**, and 22.0% for **65**, respectively. In 2021, the same group provided a facile strategy to modulate emission colors and synthesized 1,4‐azaborines **67**, **134**, and **135** with high PLQYs and color purity.^[^ ^29^ ^]^ By introducing the electron‐withdrawing imine and electron‐donating amine moieties into the reported skeleton of 1,4‐azaborine **128**, the resulting materials showed systematic hypochromic and bathochromic shifts in the emission spectra with the color ranging from deep‐blue (**67**, *λ*_max_: 461 nm) to green (**134**, *λ*_max_: 515 nm) and to yellow (**135**, *λ*_max_: 571 nm). OLED devices employing these compounds as emitters exhibited high EQEs of 19.0% for **67**, 29.2% for **134**, and 19.6% for **135**, respectively.

## 4 Conclusions and Perspectives

In this review, we have discussed the synthetic strategies, the intriguing properties and promising applications of 1,4‐azaborine‐embedded conjugated materials. The synthetic strategies for air‐stable 1,4‐BN‐heteroarenes are generally divided into the following three types: 1) transition‐metal‐catalyzed reaction, 2) nucleophilic substitution, and 3) direct electrophilic borylation. The first two strategies are mainly used for constructing monocyclic and linear 1,4‐BN‐heteroarenes, whereas the third strategy has been widely utilized to build 1,4‐BN‐PAHs with various architectures. Meanwhile, the value of incorporating 1,4‐azaborine moieties in materials has been summarized, including the modulating effect on photophysical and chiroptical properties, the roles as novel ligands for metal catalysis and as precursors for atomically precise BN‐doped graphene nanoribbons, and the applications in MR‐TADF OLEDs. These advances demonstrate a bright future of 1,4‐azaborine‐based materials.

Despite the significant achievements, there are remaining challenges and opportunities that should draw attention in the future. Firstly, most of the 1,4‐BN‐heteroarenes are built up via the electrophilic borylation strategy, which still bears certain disadvantages, such as harsh reaction conditions (usually under high temperatures) and low regioselectivity. These drawbacks somewhat hamper the diversity of 1,4‐BN‐heteroarenes. Hence, it is still necessary to develop new facile strategies for constructing 1,4‐azaborine‐based PAHs with novel conjugated skeletons. Second, although 1,4‐BN‐heteroarenes have been widely explored as MR‐TADF materials, the emission wavelength only covers the region from 460 to 692 nm, and especially, NIR emission based on 1,4‐BN‐heteroarenes has remained elusive. Therefore, there is still a broad space to tune the molecular properties of MR‐TADF materials in the future. Finally, in recent five years, 1,4‐BN‐heteroarenes have mainly been used for MR‐TADF OLEDs, whereas only few other applications have been reported. The applications of 1,4‐BN‐heteroarenes beyond OLEDs should be further explored given their unique properties. We believe that this review would stimulate more interest in the synthetic chemistry of 1,4‐azaborines and promote the future development of 1,4‐azaborine‐based materials with deeper understanding of the structure‐property relationships.

## Conflict of Interest

The authors declare no conflict of interest.
